# Crystal structures of the components of the *Staphylococcus aureus* leukotoxin ED

**DOI:** 10.1107/S2059798315023207

**Published:** 2016-01-01

**Authors:** S. Nocadello, G. Minasov, L. Shuvalova, I. Dubrovska, E. Sabini, F. Bagnoli, G. Grandi, W. F. Anderson

**Affiliations:** a Center for Structural Genomics of Infectious Diseases, Department of Biochemistry and Molecular Genetics, Northwestern University Feinberg School of Medicine, Chicago, IL 60611, USA; b Novartis Vaccines and Diagnostics, Research Centre, Siena, Italy

**Keywords:** LukE, LukD, leukotoxin, pore-forming toxins, *Staphylococcus aureus*

## Abstract

Staphylococcal leukotoxins are a family of β-barrel, bicomponent, pore-forming toxins with membrane-damaging functions. These bacterial exotoxins share sequence and structural homology and target several host-cell types. Leukotoxin ED (LukED) is one of these bicomponent pore-forming toxins that *Staphylococcus aureus* produces in order to suppress the ability of the host to contain the infection. The recent delineation of the important role that LukED plays in *S. aureus* pathogenesis and the identification of its protein receptors, combined with its presence in *S. aureus* methicillin-resistant epidemic strains, establish this leukocidin as a possible target for the development of novel therapeutics. Here, the crystal structures of the water-soluble LukE and LukD components of LukED have been determined. The two structures illustrate the tertiary-structural variability with respect to the other leukotoxins while retaining the conservation of the residues involved in the interaction of the protomers in the bipartite leukotoxin in the pore complex.

## Abstract 2

Crystal structures of LukE and LukD from *S. aureus* are reported at 3.20 and 1.70 Å resolution, respectively.

## 1. Introduction   

*Staphylococcus aureus* is a major human pathogen that has been able to rapidly acquire antibiotic resistance. The increase in the incidence of methicillin-resistant *S. aureus* (MRSA) in individuals with staphylococcal infections and the increased dominance of highly virulent strains that cause aggressive disease have diminished the success of therapeutic strategies (Marty *et al.*, 2006 ▸; Silva *et al.*, 2014 ▸). A characteristic feature of *S. aureus* pathogenesis is its ability to secrete a broad range of immune-system evasion factors. Included among these are pore-forming toxins that are of interest in the development of new therapeutic approaches (Vandenesch *et al.*, 2012 ▸; Alonzo & Torres, 2013 ▸). The pore-forming toxins are virulence factors that are expressed as water-soluble monomeric proteins. After the recognition of their receptors they assemble on the membranes of the target cells to form a pore (Nguyen *et al.*, 2003 ▸). Based on the secondary structure of the transmembrane region of the pore structure, the pore-forming toxins can be classified into two families: the α-helical family and the β-barrel family. The β-barrel family is composed of one single-component pore-forming toxin member, αHL, and three bicomponent pore-forming toxin members, γ-hemolysin (γ-HL), Panton–Valentine leukocidin (PVL) and leukocidin (Luk) (Ferreras *et al.*, 1998 ▸). To date, there are six known bicomponent leukocidins: LukSF-PV, HlgAB, HlgCB, LukAB/HG, LukED and LukMF (Alonzo & Torres, 2014 ▸). The bicomponent pore-forming toxins have two separate water-soluble subunits, one of class S (related to the slow-eluted component of PVL) and one of class F (related to the fast-eluted component of PVL).

Recently, a number of protein receptors have been added to the list of host molecules that are known to interact with the bicomponent pore-forming toxins, dramatically increasing the understanding of specific cell targeting by *S. aureus* (DuMont & Torres, 2014 ▸). Indeed, certain cellular membrane lipids are known to bind and even facilitate the prepore-to-pore transition for most leukocidins (Woodin & Wieneke, 1967 ▸; Potrich *et al.*, 2009 ▸). The S subunit is typically involved in targeting the specific cell type *via* the interaction with surface receptors and in the recruitment of the other toxin subunits, leading to oligomerization and the formation of an octameric prepore. The prepore is composed of alternating S and F subunits and is able to assemble a β-barrel pore domain in the cell membrane, producing the mature pore that ultimately induces cell death by osmotic lysis (Yamashita *et al.*, 2014 ▸).

The crystal structures of the α-hemolysin homo-heptamer, the HlgAB and LukAB hetero-octamer pore and of HlgAB and HlgBC in the hetero-octamer pre-pore state have recently been determined, revealing important details of the toxin-assembly process (Yamashita *et al.*, 2011 ▸, 2014 ▸; Song *et al.*, 1996 ▸; Badarau *et al.*, 2015 ▸). The mushroom-shaped pore complex is divided into three domains that resemble the domains of the soluble monomers: the cap, the rim and the stem. The cap consists of the β-sandwich and the latch domain from each protomer. The rim domain consists of an open-face sandwich, which extends the cap domain underneath. The rim is important for interaction with the lipid bilayer and for the interaction with specific membrane receptors. In each protomer the stem domain participates in the transmembrane β-barrel formation that ultimately perforates the membrane. The secreted water-soluble monomers reveal a similar overall structure of the protomer except for the stem region, which adopts a more compact conformation as three antiparallel β-strands stacked with the β-strands of the cap domain (Roblin *et al.*, 2008 ▸; Laventie *et al.*, 2014 ▸; Guillet *et al.*, 2004 ▸; Pédelacq *et al.*, 1999 ▸).

LukED is one of the major virulence factors that *S. aureus* uses in bloodstream infections and it plays a critical role in pathogenesis, as shown by the fact that an isogenic highly virulent staphylococcal strain with *lukED* deleted has a dramatic attenuation in animal models (Alonzo *et al.*, 2012 ▸; Reyes-Robles *et al.*, 2013 ▸). LukE targets monocytes, neutrophils, macrophages, T-cells, dendritic cells and NK cells from various species, including mice (Alonzo *et al.*, 2012 ▸, 2013 ▸; Wright, 1936 ▸; Bownik, 2006 ▸; Siwicki *et al.*, 2003 ▸). Furthermore, LukED is the only leukotoxin with a high level of neutrophil killing (Alonzo *et al.*, 2012 ▸). The broad host range of cell targeting by LukED has been partially clarified by the recent identification of CCR5, CXCR1 and CXCR2 as its binding partners (Alonzo *et al.*, 2013 ▸; Reyes-Robles *et al.*, 2013 ▸). Binding these three cellular receptors allows LukED to target both innate and adaptive immunity. Indeed, LukE and LukD exhibit almost no sequence diversity among the sequenced *S. aureus* strains (McCarthy & Lindsay, 2013 ▸; von Eiff *et al.*, 2004 ▸). Furthermore, LukED has been shown to be associated with staphylococcal bullous impetigo and post-antibiotic diarrhea (Menestrina *et al.*, 2003 ▸). These observations single out LukED as a target for alternative and more effective therapy for staphylococcal infections.

In the present study, we determined the crystal structures of both of the water-soluble components of the LukED pore-forming toxin. The structures display conservation of the core domains of LukE and LukD with the other members of the family. Furthermore, this study suggests which residues are important for CXCR1/CXCR2 binding and for the interaction of the two protomers involved in the stabilization of the pore complex. These results have the potential to aid drug discovery by targeting this important staphylococcal virulence factor.

## 2. Experimental procedures   

### 2.1. Crystallization, data collection and phasing   

*lukE* and *lukD* were amplified by PCR from *S. aureus* strain NCTC8325 and cloned into the pET-15b+ vector using the Polymerase Incomplete Primer Extension (PIPE) technique (Klock & Lesley, 2009 ▸). Standard protocols routinely implemented at the Center for the Structural Genomics of Infectious Diseases (CSGID) were used for the expression and purification of LukE and LukD (Kim *et al.*, 2008 ▸; Anderson, 2014 ▸). Following transformation into the BL21 (DE3) Magic *Escherichia coli* strain, cells were grown in TB medium at 37°C until an OD_600_ of 1 was attained. At this point, the temperature was reduced to 25°C and protein overexpression was induced by the addition of isopropyl β-d-1-thiogalacto­pyranoside to a final concentration of 1 m*M*. After 16 h, the cells were harvested by centrifugation, suspended in a buffer consisting of 10 m*M* Tris–HCl pH 8.3, 500 m*M* NaCl, 10% glycerol, 5 m*M* β-mercaptoethanol and lysed by sonication. LukE and LukD were each separately purified by Ni–NTA affinity chromatography and eluted in a buffer consisting of 10 m*M* Tris–HCl pH 8.3, 500 m*M* NaCl, 5 m*M* β-mercapto­ethanol. Immediately following their purification, the LukE and LukD proteins were concentrated to 14.2 and 5.2 mg ml^−1^, respectively, and stored in a buffer consisting of 0.5 *M* NaCl, 10 m*M* Tris–HCl pH 8.3.

Sitting-drop crystallization trials were set up at room temperature and crystals were obtained using LukE at a concentration of 7.1 mg ml^−1^ in 0.2 *M* lithium sulfate, 0.1 *M* Tris pH 8.5, 40%(*v*/*v*) PEG 400 and LukD at 2.6 mg ml^−1^ in 0.2 *M* ammonium acetate, 0.1 *M* bis-tris pH 6.5, 25%(*w*/*v*) PEG 3350. Harvested crystals were transferred to mother liquor before being rapidly cooled in liquid nitrogen. Diffraction data were collected at 100 K on the Life Sciences Collaborative Access Team beamlines at the Advanced Photon Source, Argonne, Illinois, USA. Data were processed using *HKL*-2000 for indexing, integration and scaling (Otwinowski & Minor, 1997 ▸). Structures were determined by molecular replacement using *Phaser* (McCoy *et al.*, 2005 ▸). Data-collection and refinement statistics are given in Table 1 ▸.

The structures of *S. aureus* LukS-PV and LukF-PV were used as molecular-replacement models to determine the structures of LukE and LukD, respectively. The structures were refined with *REFMAC* v.5.7.0032 (Murshudov *et al.*, 2011 ▸). Models were displayed in *Coot* and manually corrected based on electron-density maps (Emsley & Cowtan, 2004 ▸). All structure figures were prepared using *PyMOL* (v.1.3; Schrödinger). The structure of LukE also contains two Cl atoms and a molecule of triethylene glycol; the LukD structure contains a molecule of 2-[bis(2-hydroxy­ethyl)amino]-2-(hydroxymethyl)propane-1,3-diol. The sequence alignment was performed using the *ESPript*3.0 server (Robert & Gouet, 2014 ▸).

## 3. Results   

### 3.1. Structure determination of LukE and LukD   

**Figure 1:**
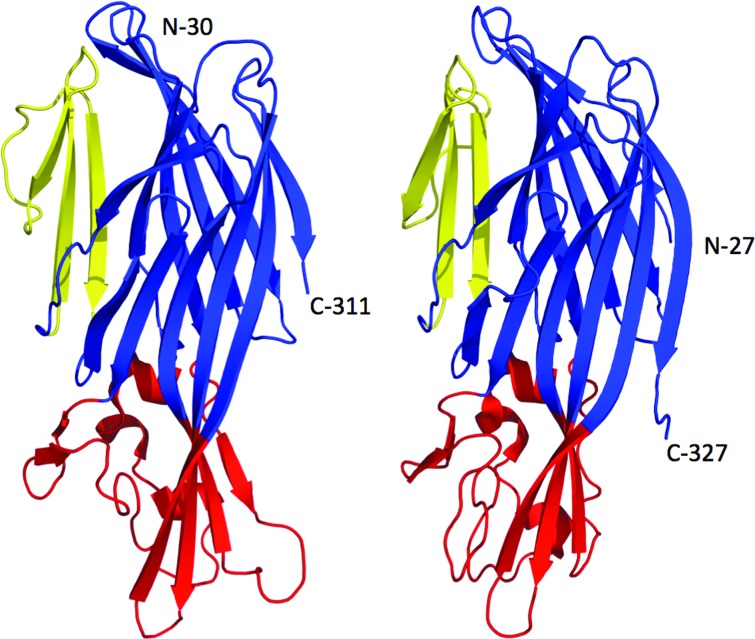
Three-dimensional structures of the two water-soluble components of the LukED pore-forming leukocidin. Ribbon representations of LukE (left) and LukD (right) with the β-sandwich of the cap, stem and rim in blue, yellow and red, respectively. The N- and C-termini are labeled.

Several constructs for LukE and LukD were tested for expression using different expression systems in *E. coli*. LukE_12–311_ and LukD_27–327_ were successfully expressed and purified and were used in crystallization screening. Crystals of LukE_12–311_ and LukD_27–327_ provided measureable X-ray diffraction data to 3.2 and 1.7 Å resolution, respectively. The structures were determined by molecular replacement using the previously determined three-dimensional coordinates of LukS-PV and LukF-PV as search models (Pédelacq *et al.*, 1999 ▸). Each of the two crystal structures had one molecule in the asymmetric unit, and the two refined models included amino acids 30–311 of a total of 311 for LukE and amino acids 27–325 of a total of 327 for LukD. LukE and LukD are each globular proteins of ellipsoidal shape with 22 (175 residues) or 25 (176 residues) β-strands, respectively, organized as four antiparallel β-sheets and three very short α-helices (ten residues). Their organization is very similar to the fold of the other bicomponent pore-forming toxins as well as to α-hemolysin and they are arranged in three typical domains: (i) cap, (ii) stem and (iii) rim (Gouaux *et al.*, 1997 ▸; Pédelacq *et al.*, 1999 ▸; Guillet *et al.*, 2004 ▸; Fig. 1 ▸). The first 30 residues of LukE and 27 residues of LukD are missing at both N-termini because the leader peptide for secretion in *S. aureus* was truncated and also because the electron density was poorly defined in this area.

### 3.2. Structure of LukE and LukD in comparison to other leukotoxins   

**Figure 2:**
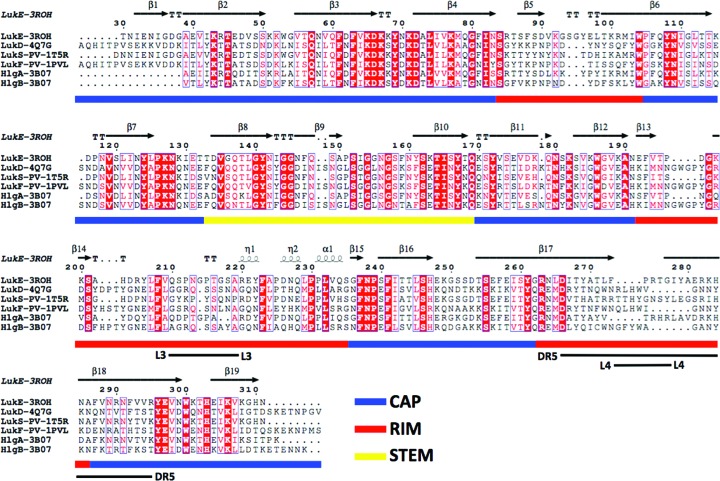
Sequence conservation of LukE and LukD with other bicomponent pore-forming toxins. The residues in each column of the multiple sequence alignment are colored according to the equivalent (in red) or to the conservation of the physical-chemical properties. The bars in blue, yellow and red indicate the regions belonging to the cap, the stem and the rim, respectively. Loops L3 and L4 and the divergent region DR5 are also indicated.

**Figure 3:**
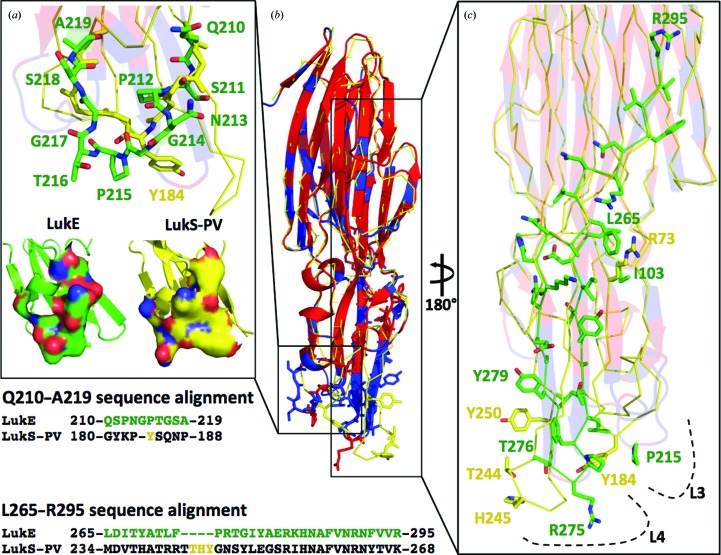
Superimposition of LukE and LukS-PV. LukE is colored by sequence conservation with LukS-PV (red, conserved; blue, nonconserved) and is superimposed onto LukS-PV (PDB entry 1t5r; solid line in yellow; Guillet *et al.*, 2004 ▸). (*a*) Top, close-up of residues Gln210–Ala219 of LukE (green) superimposed onto the main chain of LukS-PV shown in stick repesentation (yellow; the only side chain shown is that of Tyr184). Bottom, the two solvent-exposed surfaces of LukE (left, green) and LukS-PV (right, yellow). In (*b*) the amino acids of the divergent regions Gln210–Ala219 and Leu265–Arg295 (DR5) are represented as sticks in LukE. The sequence alignment of LukE and LukS-PV for Gln210–Ala219 and Leu265–Arg295 is reported at the bottom left. (*c*) The divergent region DR5 shown as green sticks in LukE, with the positions of Arg73, Tyr184, Thr244, His245 and Tyr250 of LukS-PV, which are involved in binding to hPMNs cells, neutrophil activation and pore formation, shown as yellow sticks. In LukE, these residues correspond to Ile103, Pro215, Arg275, Thr276 and Tyr279. Loops L3 and L4 are indicated in the rim domain.

It was demonstrated that LukED targets the chemokine receptors CXCR1 and CXCR2 on neutrophils (Reyes-Robles *et al.*, 2013 ▸). The interaction occurs through LukE and is inhibited by CXCL8, which is the high-affinity endogenous ligand of both CXCR1 and CXCR2. The region Gln210–Ala219 of LukE, corresponding to loop L3 (Fig. 2 ▸), confers specificity to CXCR1/CXCR2; indeed, hybrid LukE molecules harboring the LukS-PV sequence in this region are unable to target the CXCR1 and CXCR2 receptors, while still conserving the toxicity against other cell types (Fig. 3 ▸ *a*; Reyes-Robles *et al.*, 2013 ▸). The overall superimposition of LukE and LukS-PV has a larger r.m.s.d. compared with superimposition of LukE and other S members (r.m.s.d. 1.2 Å). This is owing to a higher level of torsion of the rim domain of LukS-PV with respect to the cap domain. Comparison of the surfaces of LukE and LukS-PV in the region Gln210–Ala219 shows that the amino-acid sequence divergence includes residues that contribute to the variation of the surface properties (Fig. 3 ▸ *a*). In particular, the substitution of Tyr184 of LukS-PV with Gly214 in LukE seems to have a major impact on the area of the solvent-exposed surface and on the shape of loop L3. The fact that loop L3 in LukE is one residue longer compared with the same loop in LukS-PV results in positioning residues Pro215-Thr216 so that the local solvent exposure is higher. Other residues that strongly affect the surface of L3 in LukE are Gln210 and Ser218. Indeed, the solvent-exposed surface of loop L3 is 1750.8 Å^2^ in LukE and 1472.4 Å^2^ in LukS-PV with a different charge distribution. These important variations could explain the different specificity of binding of LukE and LukS-PV to chemokine receptors (Reyes-Robles *et al.*, 2013 ▸). Furthermore, LukE and LukD exhibit little or no sequence diversity among different *S. aureus* strains and the alignment of the sequences of LukE from 150 different strains did not identify relevant variability in loop L3. Indeed, the divergence of L3 in LukE with respect to LukS-PV is not essential for the toxicity of LukED in the CCR5+ cell line, suggesting that it is not required for binding to the CCR5 receptor (Reyes-Robles *et al.*, 2013 ▸).

Another divergent region of LukE with respect to LukS-PV is the sequence Leu265–Arg295, called DR5, which is important for the toxic activity of LukED (Fig. 3 ▸ *c*; Reyes-Robles *et al.*, 2013 ▸). In this region there are residues that are important for the receptor binding of LukS-PV. Although LukS-PV shares high amino-acid identity with LukE, it uses the C5a receptor to target human PMNs. Recently, scanning mutagenesis of LukS-PV has been performed to identify residues involved in its binding to the neutrophil surface (Laventie *et al.*, 2014 ▸). In particular, single-residue mutations of Arg73, Tyr184, Thr244, His245 and Tyr250, each to alanine, as reported in Figs. 3 ▸(*a*) and 3 ▸(*c*), have been shown to reduce the ability of LukS-PV to activate neutrophils and to form the pore, and are important for the binding to human polymorphonuclear leukocytes (hPMNs). In LukE, these residues correspond to Ile103, Pro215, Arg275, Thr276 and Tyr279. In particular, the substitution of Tyr184 in LukS-PV by Pro215 in LukE seems to be particularly important in reducing (i) the distance between loops L3 and L4, resulting in a distance of 8.4 and 10.6 Å between the C^α^ atom of Tyr184 (in LukS-PV) or Pro215 (in LukE) and the C^α^ atoms of the closest residues on loop 4, (ii) the torsion of L3 itself (the distance between the C^α^ atom of Tyr184 and Pro215 is 3.2 Å upon superimposition) and (iii) the general torsion of the rim in LukE (the plane of the β-sheet of L4 rotates by ∼74° in LukS-PV with respect to LukE).

**Figure 4:**
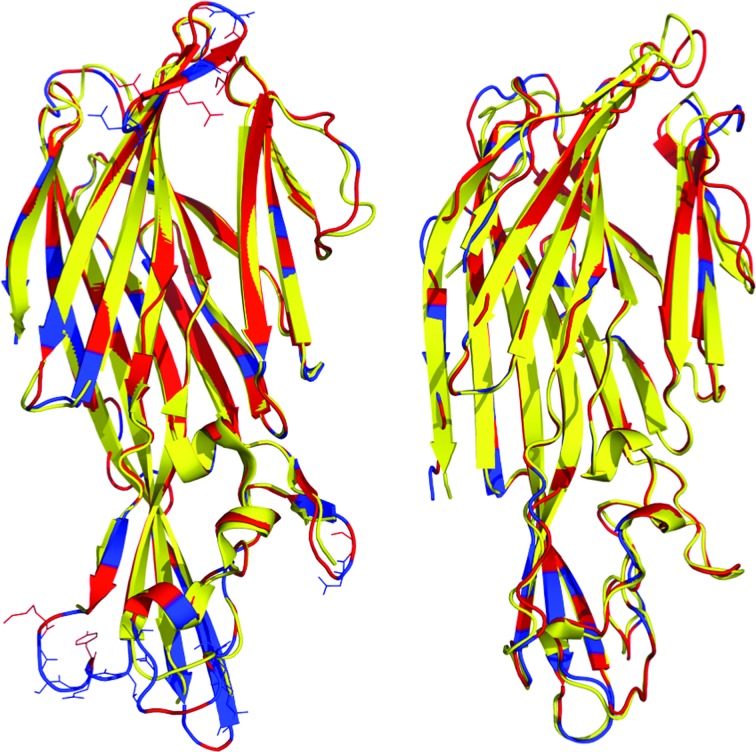
Superimposition of LukE and LukD with HlgA and HlgB. Left, ribbon representation of LukE colored by sequence conservation with respect to HlgA (red, conserved; blue, nonconserved) superimposed on HlgA (PDB entry 2qk7) in yellow. The missing regions in the structures of HlgA are indicated in the superimpositions on LukE, respectively, with the residues drawn as sticks. Right, structural superimposition of LukD with HlgB (PDB entry 1lkf). LukD is colored according to sequence conservation with HlgB.

To date, HlgA and HlgB are the only components from the bicomponent pore-forming toxins for which the structures of both the water-soluble form as well as the pore form have been determined. HlgA and HlgB share 71 and 76% identity with LukE and LukD, respectively. In Fig. 4 ▸, the water-soluble forms of HlgA and HlgB have been superimposed with LukE and LukD. LukE–HlgA superimposition results in an r.ms.d. of 0.53 Å. In part, this value is so low because the residues that are likely to be most variable are missing in the HlgA structure. The highest variability (shown by the residues drawn as sticks in Fig. 4 ▸) is observed in the loops of the rim and in the latch domain of the cap, which is not present in the structure of HlgA (PDB entry 2qk7; Roblin *et al.*, 2008 ▸). Furthermore, LukD–HlgB superimposition results in an r.m.s.d. of 0.97 Å. The structural variability is observed primarily in the loop connecting two antiparallel β-strands in the compact conformation of the stem that is not present in the structure of HlgB (PDB entry 1lkf; Olson *et al.*, 1999 ▸). The phosphocholine-binding pocket that is occupied in HlgB by a molecule of MPD in the pore structure or by a molecule of phosphocholine in the structure of the water-soluble monomer is occupied by a molecule of bis-tris buffer in LukD.

### 3.3. Structural features of LukE and LukD in the context of the pore-forming complex   

**Figure 5:**
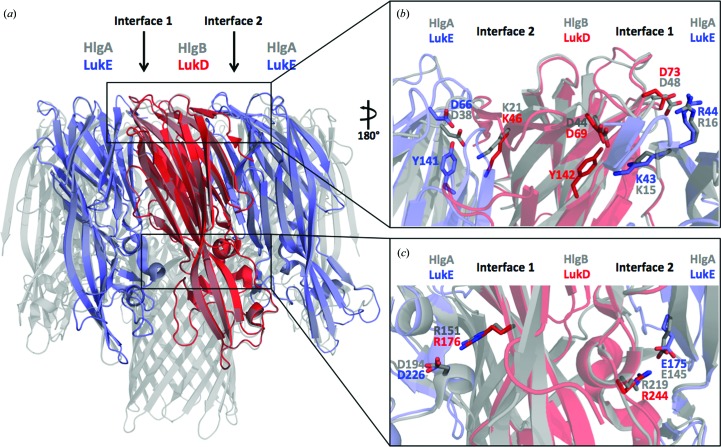
Structural superimposition of LukE and LukD on the protomers of the HlgAB pore complex. (*a*) Superimposition of LukE (blue) and LukD (red) on the protomers HlgA and HlgB, respectively, in the pore complex (PDB entry 3b07, gray; Yamashita *et al.*, 2011 ▸) reveals interface 1 and interface 2. (*b*) Interprotomer electrostatic interactions in the cap. Residues from HlgA and HlgB that form electrostatic interaction (gray sticks) are conserved in LukE (slate sticks) and LukD (red sticks). (*c*) Interprotomer electrostatic interactions in the rim. The conserved residues from HlgA and HlgB that form electrostatic interactions in the pore complex are superimposed in LukE and LukD. Sticks are colored as in (*a*).

Recently, the structures of the γHL and LukAB octameric pores have been determined (Yamashita *et al.*, 2011 ▸, 2014 ▸; Badarau *et al.*, 2015 ▸). The octamers are formed by four molecules of each of the S and F subunits. Furthermore, it has been suggested that the octameric structure will resemble the pore structure of the other leukocidins (Alonzo & Torres, 2014 ▸). Since it has been shown that the core of the cap and the rim domains are rigid between the monomer and protomer in each member of γHL and that they conserve their relative orientation upon octamer assembly, the structures of LukE and LukD have been superimposed on HlgA and HlgB of the γHL pore complex (LukE, r.m.s.d. of 1.67 Å on 232 C^α^ atoms; LukD, r.m.s.d. of 1.66 Å on 246 C^α^ atoms). We investigated the conservation of residues involved in the interactions occurring in the two types of interface between the protomers (interface 1, HlgA–HlgB, corresponding to LukE–LukD; interface 2, HlgB–HlgA, corresponding to LukD–LukE; Fig. 5 ▸). In interface 1 of the cap domain, Asp44 and Asp48 of HlgB and Lys15 and Arg16 on HlgA are involved in the interprotomer electrostatic interaction. These residues are conserved and correspond to Asp69 and Asp73 of LukD and Lys43 and Arg44 of LukE. In interface 1 of the rim domain, Arg171 of HlgB and Asp194 of HlgA are involved in the electrostatic interaction and correspond to Arg 176 of LukD and Asp226 of LukE. Furthermore, Asp66 and Asp175 of LukE and Lys46 and Arg244 of LukD, corresponding to Asp38 and Glu145 of HlgA and Lys21 and Arg219 of HlgB, are involved in the interprotomer electrostatic interaction on interface 2. In addition, Asp66 of LukE and Asp69 of LukD seem to also be very important in stabilizing the stems in the soluble form. Indeed, they are involved in intra­protomer hydrogen-bond interactions with Tyr141 and Tyr142 in the stems in LukE and in LukD, respectively, as well as with the main chain of the stems, indicating the importance of these residues in the transition of the soluble monomers to the pore-complex state (Supplementary Fig. S1).

The stem region undergoes the most important structural remodeling between the monomers and the protomers (Roblin *et al.*, 2008 ▸). Besides the hydrogen bonds that occur in the interprotomer β-sheets, two ion pairs between Glu108 and Lys146 of HlgB and Lys140 and Asp104 of HlgA are found in the γHL octameric pore. According to the sequence alignment, they correspond to Glu133 and Lys171 of LukD and Lys170 and Asp134 of LukE.

## 4. Discussion   

The β-barrel pore-forming toxins of the *S. aureus* leukocidin family are important for many aspects of staphylococcal infection, including involvement in serious human pathologies. Understanding the biochemical properties of the water-soluble members and their structural remodeling during formation of the pore complex will aid in the development of new, efficient strategies to control staphylococcal infection. Despite this class of toxins having been identified more then a century ago, efforts during the last five years have provided new insight into their molecular mechanism of action in the context of pore assembly, receptor identification and the general mechanism of immune escape (Alonzo & Torres, 2013 ▸, 2014 ▸). In particular, LukED has been the subject of revived interest during the last few years because of its high association with virulent strains, including the MRSA strains responsible for the current pandemic (Alonzo *et al.*, 2012 ▸). Furthermore, they have a high level of conservation among *S. aureus* strains and a unique toxicity toward human neutrophils, rabbit red blood cells and mouse phagocytes; furthermore, their toxicity to mouse phagocytes correlates with the lethality observed in the mouse model of bacteremia (Alonzo *et al.*, 2012 ▸).

In this context, the crystal structures of LukE and LukD in the water-soluble state that we report here will be of interest. The broad activity of LukED on a wide variety of cell types from various species has recently been explained by the identification of the binding receptors of LukED. Indeed, LukE targets CCR5 to kill inflammatory macrophages, dendritic cells and T cells (Alonzo *et al.*, 2013 ▸). Furthermore, LukE binds CXCR1 and CXCR2 and previous studies have identified the L3 loop in the rim domain as the binding region (Reyes-Robles *et al.*, 2013 ▸). The crystal structure of LukE provides insight into the residues required for receptor identification. We argue that the structural features that determine the binding of LukE to CXCR1/CXCR2 receptors in contrast to LukS-PV are dependent on the presence of glycine and proline residues in loop L3 (Pro212, Gly214, Pro215 and Gly217) as well as a different distribution of polar residues (Gln210, Asn213, Thr216 and Ser211). Furthermore, the structure of LukE shows that key residues in loop L4 (Pro210 and Pro215) may have an impact on the changed position of loop L3 with respect to the overall protein, resulting in a more compact organization of L3–L4 in LukE compared with the same region in LukS-PV.

Recently, there has also been interest in elucidating the structural properties of the oligomer complex of the bi­component pore toxin. Crystal structures from the γ-hemolysin family and of LukAB are available (Yamashita *et al.*, 2011 ▸, 2014 ▸; Badarau *et al.*, 2015 ▸). The superimposition of LukE and LukD on the HlgAB complex reveals conservation of the residues that are important in the electrostatic interaction of the protomers in the complex. Furthermore, the recently determined structure of the HlgAB_HlgB-Y177A/R198A_ mutant and HlgCB in the prepore-state oligomer have delineated a model in which a two-step β-barrel formation mechanism explains the pore-formation process without the stereochemical hindrance of the previous models (Yamashita *et al.*, 2011 ▸, 2014 ▸). This model has been suggested to be valid for the entire bicomponent pore-forming toxin family, and future studies of other members of the superfamily will test its validity.

Our increased knowledge of leukocidin diversity clarifies the biochemical features defining their specificity for host immune cells. In particular, it indicates a new rational approach to target staphylococcal leukocidins in order to improve the efficacy of the treatment strategy. This may include the use of antibodies and new drugs that can inhibit toxin binding to the recently identified toxin receptors, blocking the initial interaction with the cell membrane and/or the assembly of the pore complex, all of which represent very attractive fields of investigation. Further studies will benefit from an increased biochemical/structural understanding of the mechanism of action and a more in-depth knowledge of the structural features of each member of the bicomponent pore-forming toxins.

## Supplementary Material

PDB reference: LukD, 4q7g

PDB reference: LukE, 3roh

Supplementary Figure S1.. DOI: 10.1107/S2059798315023207/dw5151sup1.pdf

## Figures and Tables

**Table 1:** Data-collection and refinement statistics for structure determinations. Values in parentheses are for the outer shell.

	LukE (PDB code 3roh)	LukD (PDB code 4q7g)
Data statistics		
Space group	*I*4	*P*2_1_2_1_2_1_
Unit-cell parameters (Å, °)	*a* = *b* = 133.9, *c* = 64.5, α = β = γ = 90.00	*a* = 49.74, *b* = 49.9, *c* = 134.9, α = β = γ = 90.00
Resolution range (Å)	30.00–3.20 (3.26–3.20)	30.00–1.70 (1.73–1.70)
*R*_merge_(*I*)	0.077 (0.552)	0.067 (0.615)
〈*I*/σ(*I*)〉	28.4 (4.0)	22.9 (3.0)
Completeness (%)	99.94 (100)	99.77 (99.99)
Multiplicity	7.5 (7.6)	6.0 (5.9)
No. of reflections	9573 (467)	37802 (1897)
Refinement statistics		
Resolution range (Å)	29.94–3.20	27.95–1.70
*R*_work_/*R*_free_	0.178/0.224	0.169/0.196
Molecules in asymmetric unit	1	1
No. of protein atoms	2248	2408
No. of ligand atoms	12	14
No. of solvent atoms	26	381
R.m.s. deviations		
Bond lengths (Å)	0.008	0.010
Bond angles (°)	1.36	1.41
Ramachandran plot, residues in (%)		
Most favored regions (A, B, C)	84.1	89.4
Additional allowed regions (a, b, l, p)	15.9	10.2
Generally allowed regions (~a, ~b, ~l, ~p)	0	0.4
Disallowed regions	0	0
