# Supplementary material for: Crystal structures of the components of the Staphylococcus aureus leukotoxin ED
Source: Acta Crystallogr D Struct Biol. 2016 Jan 1;72(Pt 1):113–20. doi: 10.1107/S2059798315023207 (PMC4756620; doi:10.1107/S2059798315023207)
Supplement: Supplementary file 1 [file d-72-00113-sup1.pdf]

STRUCTURAL BIOLOGY

Volume 72 (2016)

Supporting information for article:

Crystal structures of the components of the *Staphylococcus aureus* leukotoxin ED

S. Nocadello, G. Minasov, L. Shuvalova, I. Dubrovska, E. Sabini, F. Bagnoli, G. Grandi and W. F. Anderson

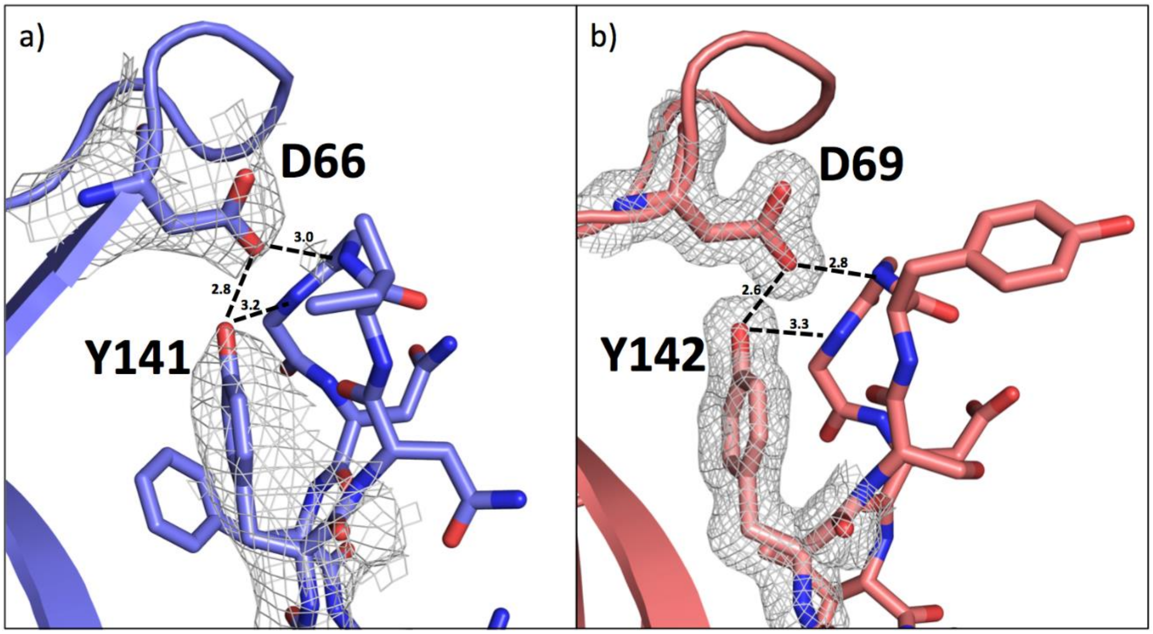

Figure S1 Particular of the hydrogen bonds of Asp66 of LukE and Asp69 of LukD with the stems in the soluble conformation. The electron-density maps contoured at 1 σ level for Asp66 and Tyr141 of LukE (a) and for Asp69 and Tyr142 of LukD (b) are shown. Asp66 and Asp69 and the stem domains are represented as sticks while the rest of the structures are represented as ribbon. It also includes the measurements of the distances between the atoms that are involved in the hydrogen bonds.
